# Supplementary material for: Inner lumen proteins stabilize doublet microtubules in cilia and flagella
Source: Nat Commun. 2019 Mar 8;10:1143. doi: 10.1038/s41467-019-09051-x (PMC6408466; doi:10.1038/s41467-019-09051-x)
Supplement: Supplementary file 1 — Supplementary Information [file 41467_2019_9051_MOESM1_ESM.pdf]

Supplementary Information:

Inner lumen proteins stabilize doublet microtubules in cilia and flagella.

Owa et al.

Supplementary Figures and Legends

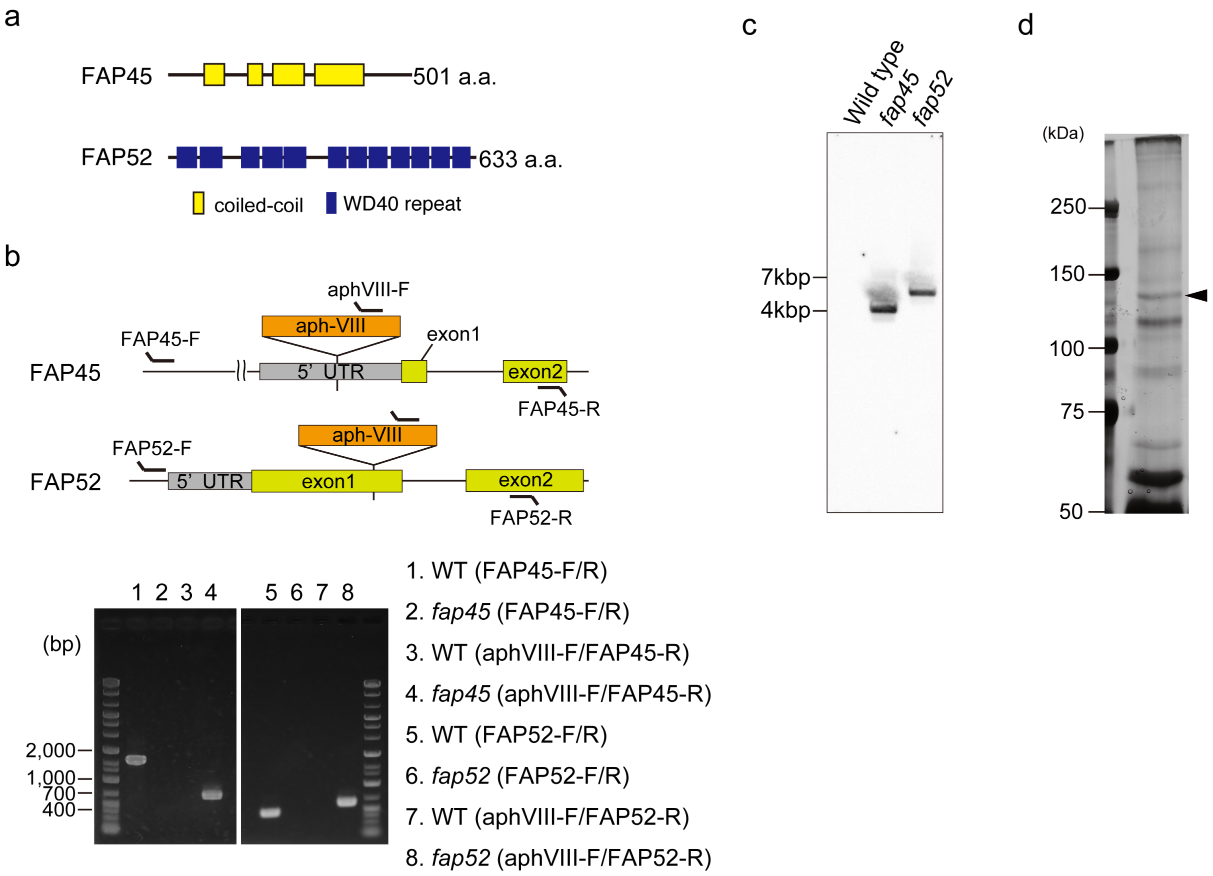

Supplementary Figure 1. Further characterizations of FAP45, FAP52 and the mutants. (a) Prediction of the structural motifs of FAP45 and FAP52. (b) The insertional mutation sites of *fap45* and *fap52* are indicated on each exon/intron structure. PCR using the aph-VIII primer only amplified genomes extracted from the mutants (4 and 8). The primer sets surrounding the insertion sites did not amplify the mutant genomes (2 and 6) probably due to the big insertion. (c) The southern blot against SacII digested DNA fragments from wild type, *fap45* and *fap52*. The fragments containing the insertion were probed by the coding sequence of the aph-VIII gene. (d) Silver staining of FAP45-IP products (EDC-crosslinked). The band pointed by arrowhead was analyzed by the mass spec.

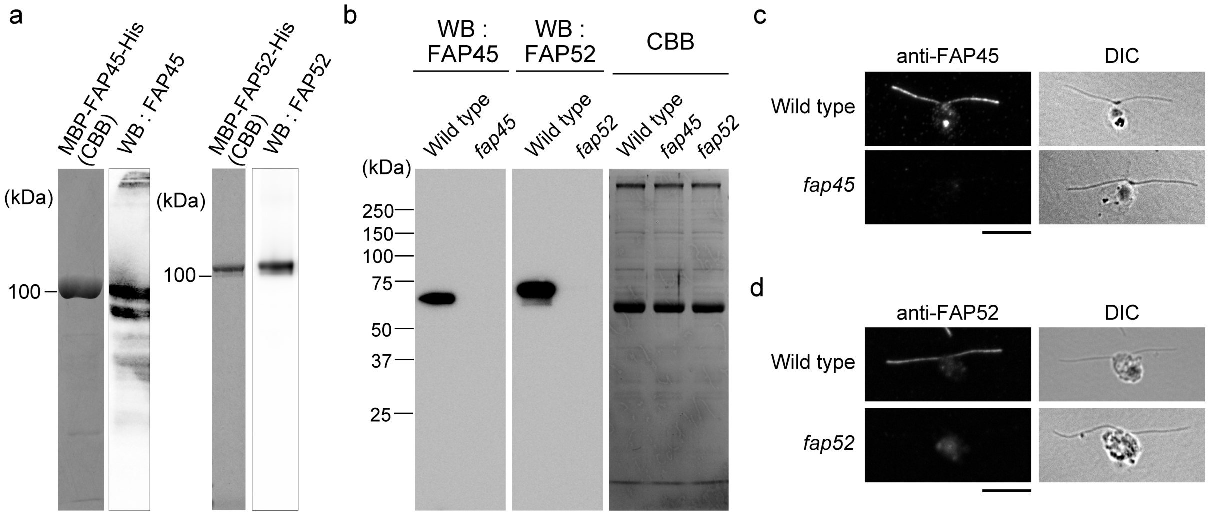

Supplementary Figure 2. *fap45* and *fap52* are null mutants. (a) CBB staining of recombinant MBP-FAP45-His and MBP-FAP52-His used for the antigens and western blot analyses against the proteins. Lower bands in the FAP45 western blot were reaction against degraded proteins. (b) Western blots of axonemes with the anti-FAP45 and anti-FAP52 antibodies. Both antibodies specifically stain the target proteins. (c, d) Immunofluorescence microscopy of the nucleoflagellar apparatus (NFA) stained with the anti-FAP45 antibody (c) and anti-FAP52 antibody (d). Both proteins are distributed along the entire length of wild type axonemes, whereas there is no signal in the mutants. Scale bar = 10 μm.

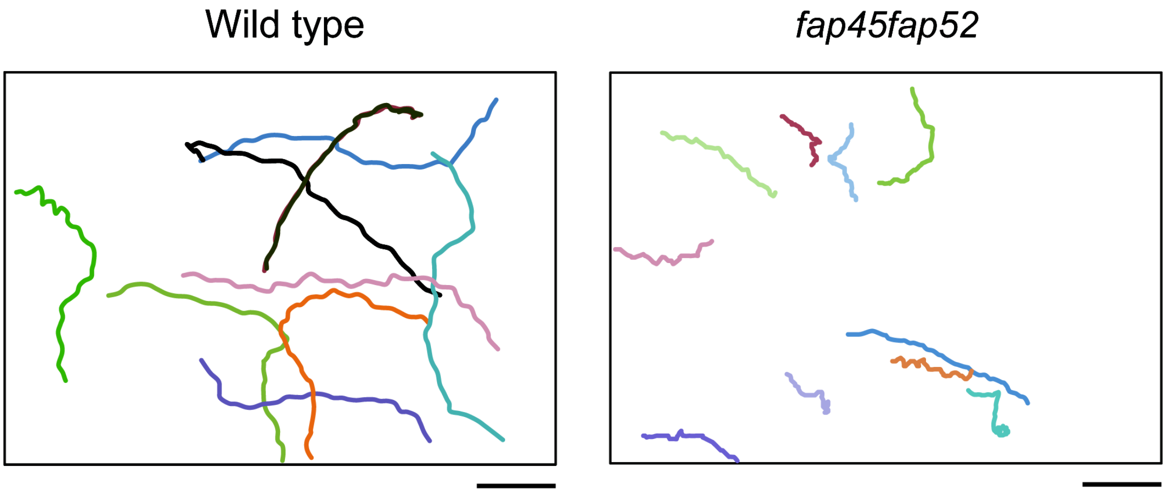

Supplementary Figure 3. Trajectories of wild type and *fap45fap52* swimming cells in TAP with 7.5% ficol. Wild type and *fap45fap52* swimming cells in TAP with 7.5% ficol were tracked for 5.5 seconds. Scale bar = 100 μm.

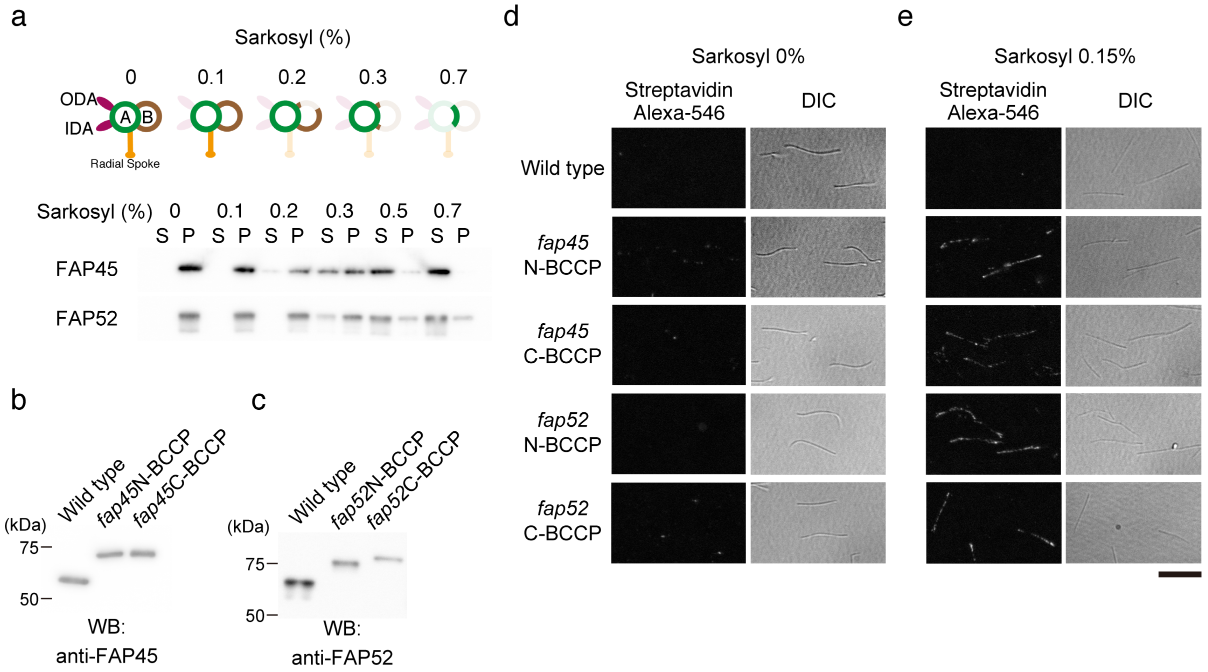

Supplementary Figure 4. FAP45 and FAP52 are luminal proteins in the B-tubule. (a) Schematic diagrams of the remaining structure of DMT after treatment with various concentrations of sarkosyl. (b) Wild type axonemes were treated with various concentrations of sarkosyl. After centrifugation, the supernatant (S) and precipitate (P) were analyzed by western blot with anti-FAP45 and anti-FAP52 antibodies. (c) Western blot of BCCP tagged FAP45 and FAP52. FAP45 and FAP52 in wild type and BCCP-tagged rescue strains were blotted with anti-FAP45 antibody or anti-FAP52 antibody. (d, e) Axonemes from wild type and BCCP-tagged rescue strains were stained with streptavidin-Alexa546 (d: intact axonemes, e: axonemes treated with 0.15% sarkosyl, DIC: differential interference contrast image, scale bar = 10 μm).

Supplementary Figure S5, Owa et al.

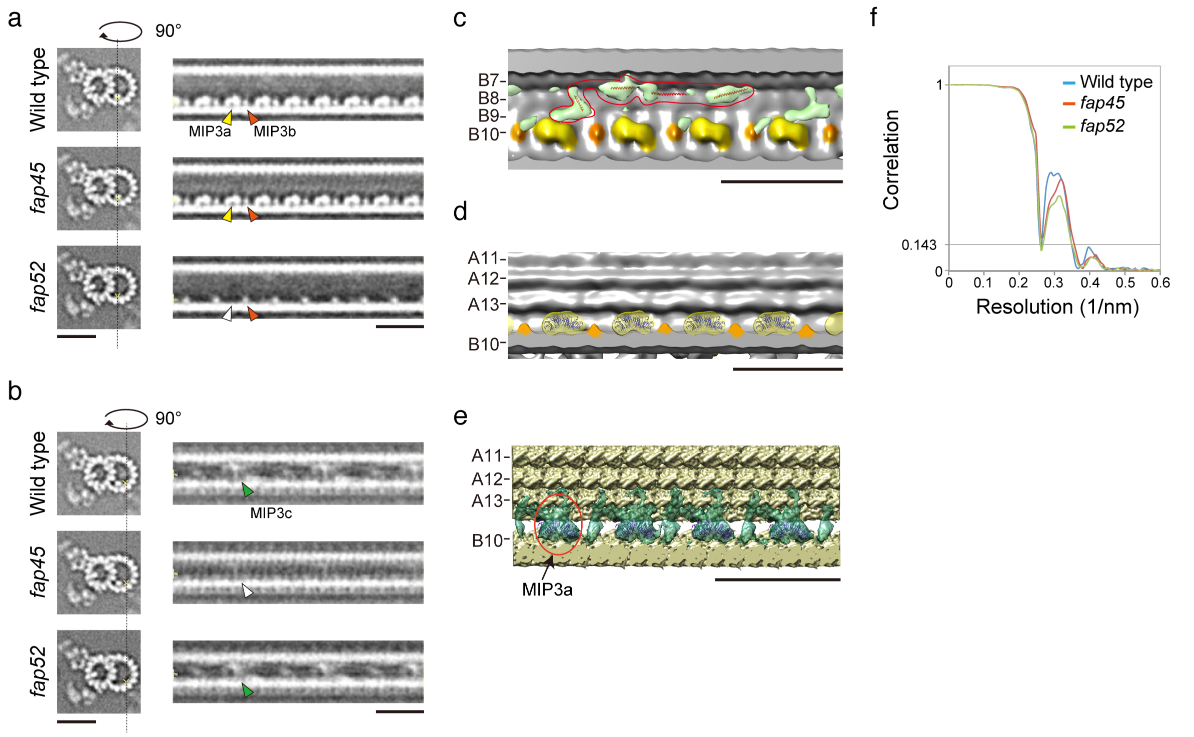

Supplementary Figure 5. Supporting data of cryo-ET. (a, b) Density maps of tomographic slices focused on MIP3a and b (a) and MIP3c (b). Scale bar = 25 nm. (c-e) Crystal structures of the coiled coils (PDB: 1DEB, modified) and the two β-propellers (PDB: 5H1M) were fit into the MIP3c (c) and MIP3a (d) densities, respectively. The two β-propellers were also fit into MIP3a of the sub-nanometer resolution map of *Tetrahymena* (e, adapted from figure 3E from Ichikawa, M. et al (2017)[22] DOI: 10.1038/ncomms15035). The *t*-value maps of MIP3a and c were acquired by the Student's *t*-tests, as previously described[23]. The maps circled in red are probable one unit of MIP3c. Scale bar = 25 nm. (f) Fourier shell correlation curves of averaged tomograms used in this study (Gold Standard; wild type, *fap45*, and *fap52*). The intersection between each curve and the horizontal line at 0.143 was taken as the effective resolution, which is 2.9 nm (wild type and *fap52*) and 2.8 nm (*fap45*).

a FAP45

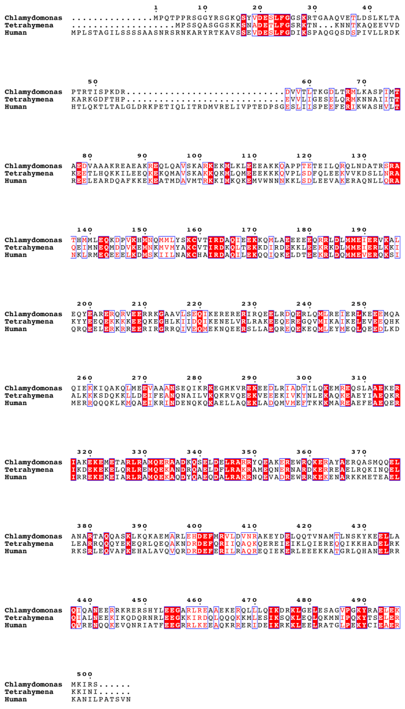

b FAP52

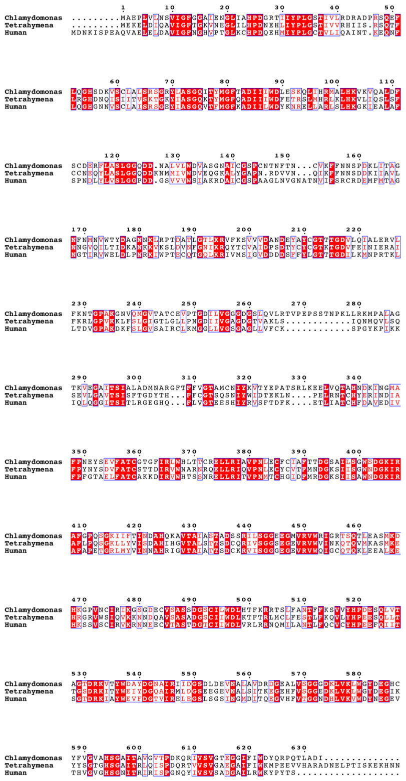

Supplementary Figure 6. Sequence comparison of FAP45 and FAP52 proteins and their orthologs. *Chlamydomonas* FAP45 (a) and FAP52 (b) were aligned with their homologs in human and *Tetrahymena* using ClustalW (http://www.ddbj.nig.ac.jp/index-j.html). Conserved residues are highlighted with the red box, and similar residues are surrounded by a blue frame. The figures were made using ESPript 3.0 (http://espript.ibcp.fr/ESPript/cgi-bin/ESPript.cgi).

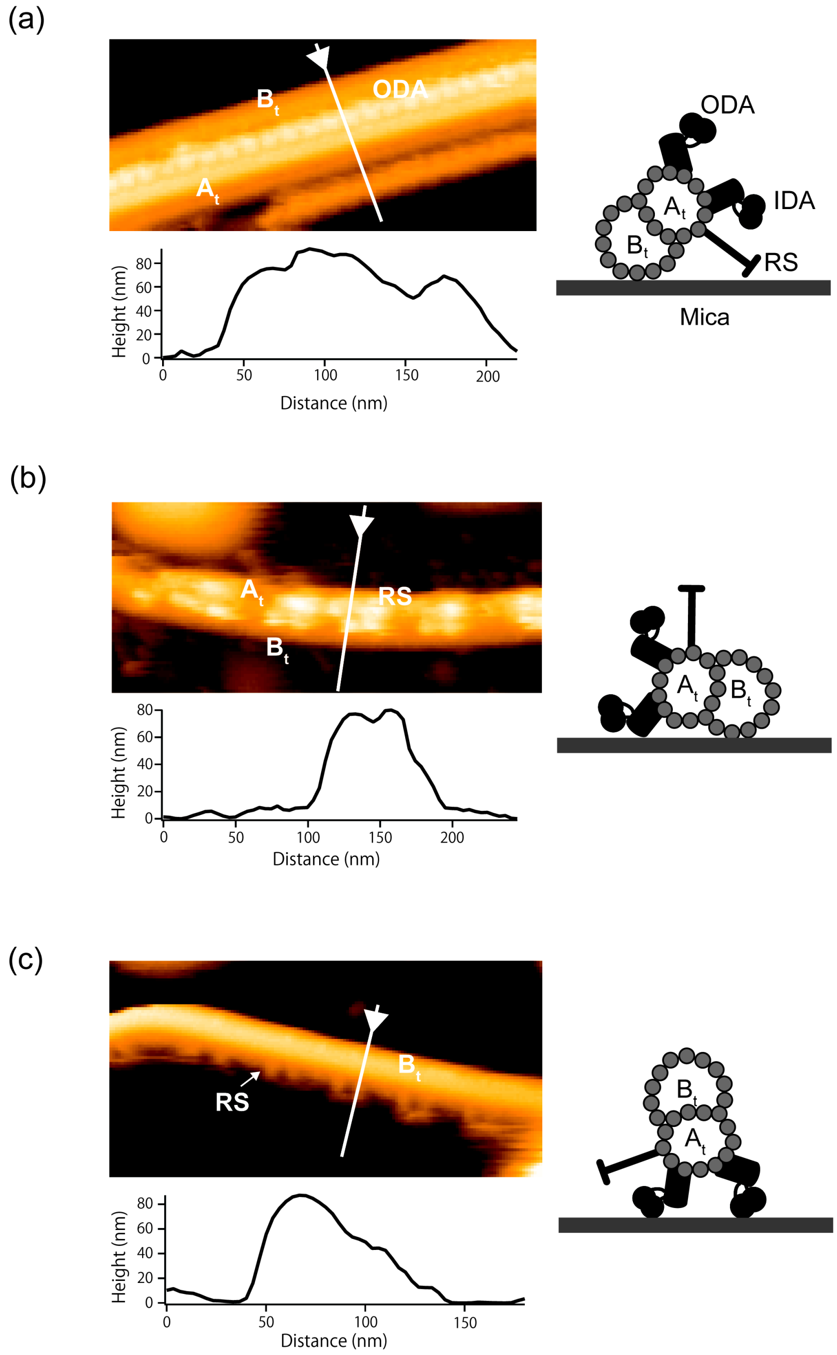

Supplementary Figure 7. Typical AFM images of DMTs. Typical AFM images of wild type-DMTs on mica substrate, corresponding cross-sectional heights along white lines and illustrations of most probable orientations of the DMTs. A_t, B_t, ODA, IDA, and RS correspond to A-tuble, B-tuble, outer-dynein arm, inner-dynein arm, and radial spoke, respectively.

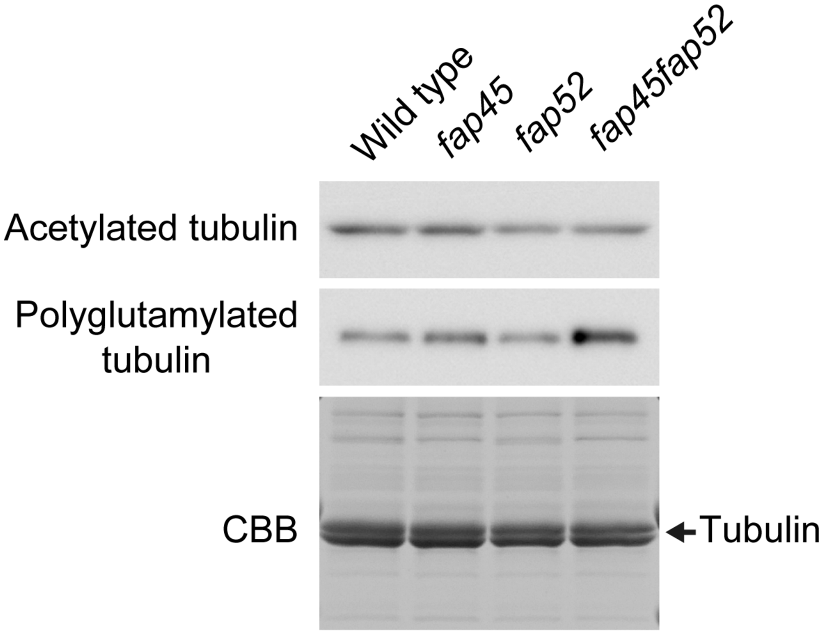

Supplementary Figure 8. Post-translational modification of tubulin in *fap45*, *fap52*, and *fap45fap52*. Axonemes of wild type, *fap45*, *fap52*, and *fap45fap52* were blotted with anti-acetylated or anti-polyglutamylated tubulin antibody. CBB staining of tubulin is used as a loading control. No significant changes in tubulin acetylation or polyglutamylation were observed in the mutants.

Figure 1

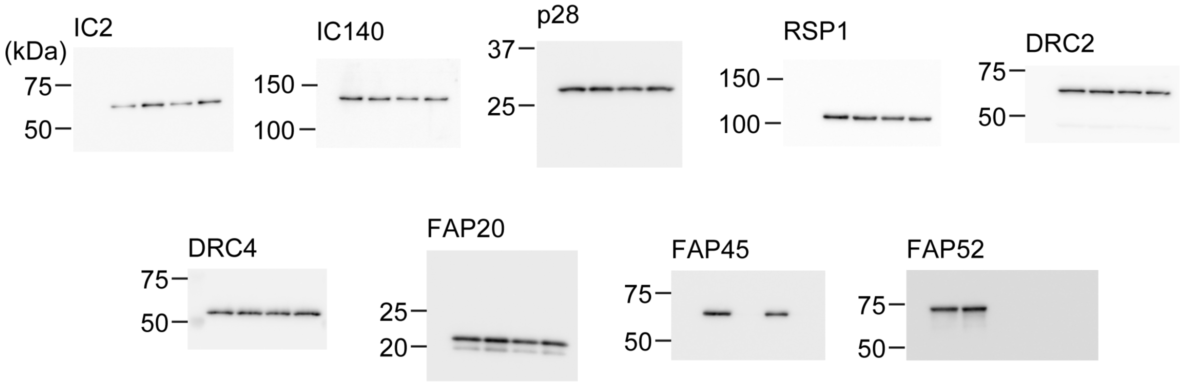

Supplementary Figure 4

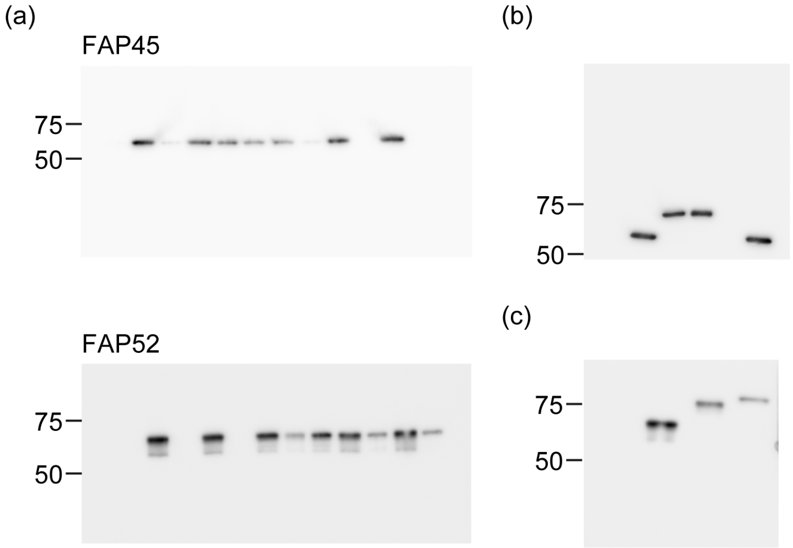

Supplementary Figure 8

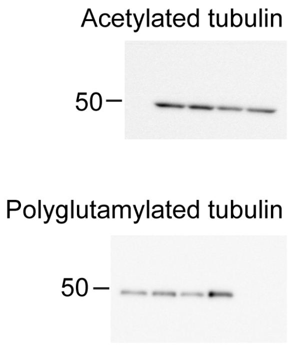

Supplementary Figure 9. **Full western blot images in this study.**

Supplementary Table 1: The *Chlamydomonas* flagellar proteome

Name	Localization	Total Unique	M+M	Axo	KCl	Reference
HYD3	Central pair	69	0	54	6	1
CPC1	Central pair	56	0	46	10	2
FAP42		54	2	45	6	Kinase?
RIB72	A-tubule	44	5	38	8	3,4
FAP46	Central pair	38	0	37	0	5
PF6	Central pair	42	0	36	5	6
FAP47	Central pair?	35	0	31	1	7
FAP50	N-DRC	31	0	31	1	8
FAP45		39	1	30	5	This study
RIB43a	A-tubule	33	0	29	0	9
FAP52		30	0	28	5	This study
FAP51		31	0	27	0	
FAP55		27	0	27	0	
FAP54		27	0	27	0	5
RSP3	Radial Spoke	27	1	26	4	10
FAP53		28	0	25	1	
DHC9		28	3	25	6	
MBO2	B-tubule?	26	0	25	0	11
FAP252		29	4	24	7	
FAP59		26	1	23	4	
FAP58		26	0	23	5	
FAP61	CSC	25	0	23	4	12
FAP63		23	0	23	0	
RSP2	Radial Spoke	25	3	21	10	13
Tektin	Close to IJ?	24	0	21	1	14
PACRG	Close to IJ?	24	0	19	6	14
RSP16	Radial Spoke	26	4	18	4	15
FAP69		20	0	18	0	
FAP7		20	2	17	9	
FAP76		18	0	17	1	
FAP250	N-DRC	21	0	16	3	16
FAP68		20	0	16	0	
FAP74	Central pair	18	0	15	0	5
HSP70		15	7	15	9	
FAP67		20	5	14	2	

FAP82		16	0	14	0	
FAP251	CSC	16	0	14	1	12
FAP77		17	0	13	5	
FAP81		16	0	13	0	
PF2	N-DRC	16	0	13	3	16
RSP23	Radial Spoke	13	0	13	1	13
RSP9	Radial Spoke	17	2	12	4	13
FAP86		15	0	12	0	
RSP5	Radial Spoke	15	5	12	5	13
RSP17	Radial Spoke	15	0	12	0	15
FAP95		14	0	12	0	
FAP71		19	0	11	3	
FAP92		15	0	11	0	
FAP85	A-tubule	15	2	11	5	17
FAP84		15	0	11	9	
FAP104		13	0	11	4	
FAP106		13	0	11	1	
FAP112		12	0	11	4	
FAP113		12	2	11	7	18
DHC4		12	0	11	0	

CSC: Calmodulin- and Radial-Spoke-Associated Complex

Supplementary Table 2: Peptides from the 130 kDa crosslinked product identified by LC MS/MS

Protein	Mascot Score	Matches	Peptide
FAP45	243	6	K.ALEQYEAR.E
			R.QASMQQELANAR.T
			K.QAPPTETEILQR.Q
			R.IRQEELRDQER.L
			K.YKEELLAQIQANEER.R
FAP52	198	4	R.TIIYPLGSTIVLR.D
			R.KVTYWDAYDGNAIR.I
			K.SVVYHPDESQLVTAGTDR.K

Supplementary Table 3: *Chlamydomonas* strains

Strain	Phenotype	Mutated gene	Reference
CC124	Wild type		
CC125	Wild type		
fap20	Trembling	FAP20	[14]
fap45	Slightly slow	FAP45	This study
fap52	Wild type	FAP52	This study
fap45fap52	slow, paralyzed		This study
fap20fap45	Trembling		This study
fap20fap52	Paralyzed		This study

Supplementary Table 4: Antibodies

Antibody	Host	Dilution	Reference
Anti-RSP1	Rabbit	1:10000 (WB)	[15]
Anti-IC2 (1869A)	Mouse	1:10000 (WB)	D6168 (Sigma-Aldrich)
Anti-IC140	Rabbit	1:5000 (WB)	[19]
Anti-p28	Rabbit	1:10000 (WB)	[20]
Anti-DRC2	Rabbit	1:5000 (WB)	[21]
Anti-DRC4	Rabbit	1:5000 (WB)	[21]
Anti-FAP20	Rabbit	1:5000 (WB)	[14]
Anti-FAP45 #1	Rabbit	1:3000 (WB)	This Study
Anti-FAP45 #2	Guinea pig	1:200 (IF)	This Study
Anti-FAP52 #2	Rabbit	1:5000 (WB), 1:200 (IF)	This Study

Anti-acetylated a-tubulin (6-11B-1)	Mouse	1:5000 (WB)	T7451 (Sigma-Aldrich)
Anti-polyglutamylated tubulin (GT335)	Mouse	1:10000 (WB)	AG-20B-0020 (Adipogen)

Supplementary References

1. Lechtreck, K.-F. & Witman, G.B. Chlamydomonas reinhardtii hydin is a central pair protein required for flagellar motility. The Journal of cell biology 176, 473-482 (2007).
2. Mitchell, D.R. & Sale, W.S. Characterization of a Chlamydomonas insertional mutant that disrupts flagellar central pair microtubule-associated structures. The Journal of cell biology 144, 293-304 (1999).
3. Ikeda, K. et al. Rib72, a conserved protein associated with the ribbon compartment of flagellar A-microtubules and potentially involved in the linkage between outer doublet microtubules. Journal of Biological Chemistry 278, 7725-7734 (2003).
4. Stoddard, D. *et al.* Tetrahymena RIB72A and RIB72B are Microtubule Inner Proteins in the ciliary doublet microtubules. *Molecular biology of the cell*, mbc. E18-06-0405 (2018).
5. Brown, J.M., DiPetrillo, C.G., Smith, E.F. & Witman, G.B. A FAP46 mutant provides new insights into the function and assembly of the C1d complex of the ciliary central apparatus. J Cell Sci 125, 3904-3913 (2012).
6. Wargo, M.J., Dymek, E.E. & Smith, E.F. Calmodulin and PF6 are components of a complex that localizes to the C1 microtubule of the flagellar central apparatus. Journal of cell science 118, 4655-4665 (2005).
7. Ponting, C.P. A novel domain suggests a ciliary function for ASPM, a brain size determining gene. *Bioinformatics* **22**, 1031-1035 (2006).

8. Bower, R. et al. The N-DRC forms a conserved biochemical complex that maintains outer doublet alignment and limits microtubule sliding in motile axonemes. Molecular biology of the cell 24, 1134-1152 (2013).

9. Norrander, J.M., Brown, J.A., Porter, M.E. & Linck, R.W. The Rib43a Protein Is Associated with Forming the Specialized Protofilament Ribbons of Flagellar Microtubules in Chlamydomonas. Molecular biology of the cell 11, 201-215 (2000).

10. Wirschell, M. *et al.* Building a radial spoke: flagellar radial spoke protein 3 (RSP3) is a dimer. *Cell motility and the cytoskeleton* **65**, 238-248 (2008).

11. Segal, R.A., Huang, B., Ramanis, Z. & Luck, D. Mutant strains of Chlamydomonas reinhardtii that move backwards only. *The Journal of cell biology* **98**, 2026-2034 (1984).

12. Urbanska, P. *et al.* The CSC proteins FAP61 and FAP251 build the basal substructures of radial spoke 3 in cilia. *Molecular biology of the cell* **26**, 1463-1475 (2015).

13. Kohno, T., Wakabayashi, K.i., Diener, D.R., Rosenbaum, J.L. & Kamiya, R. Subunit interactions within the Chlamydomonas flagellar spokehead. *Cytoskeleton* **68**, 237-246 (2011).

14. Yanagisawa, H. -a. et al. FAP20 is an inner junction protein of doublet microtubules essential for both the planar asymmetrical waveform and stability of flagella in Chlamydomonas. Molecular biology of the cell 25, 1472-1483 (2014).

15. Yang, P. et al. Radial spoke proteins of Chlamydomonas flagella. Journal of cell science 119, 1165-1174 (2006).

16. Oda, T., Yanagisawa, H. & Kikkawa, M. Detailed structural and biochemical characterization of the nexin-dynein regulatory complex. *Molecular biology of the cell* **26**, 294-304 (2015).

17. Kirima, J. & Oiwa, K. Flagellar-associated protein FAP85 is a microtubule inner protein that stabilizes microtubules. *Cell structure and function* **43**, 1-14 (2018).

18. Kamiya, R., Shiba, K., Inaba, K. & Kato-Minoura, T. Release of Sticky Glycoproteins from Chlamydomonas Flagella During Microsphere Translocation on the Surface Membrane. *Zoological science* **35**, 299-305 (2018).

19. Oda, T., Yanagisawa, H., Kamiya, R. & Kikkawa, M. A molecular ruler determines the repeat length in eukaryotic cilia and flagella. Science 346, 857-860 (2014).

20. LeDizet, M. & Piperno, G. The light chain p28 associates with a subset of inner dynein arm heavy chains in Chlamydomonas axonemes. Molecular biology of the cell 6, 697-711 (1995).

21. Oda, T., Yagi, T., Yanagisawa, H. & Kikkawa, M. Identification of the outer-inner dynein linker as a hub controller for axonemal dynein activities. Current Biology 23, 656-664 (2013).

22. Ichikawa, M. *et al.* Subnanometre-resolution structure of the doublet microtubule reveals new classes of microtubule-associated proteins. *Nature communications* **8** (2017).

23. Oda, T. & Kikkawa, M. Novel structural labeling method using cryo-electron tomography and biotin–streptavidin system. *Journal of structural biology* **183**, 305-311 (2013).
