# Supplementary material for: Inner lumen proteins stabilize doublet microtubules in cilia and flagella
Source: Nat Commun. 2019 Mar 8;10:1143. doi: 10.1038/s41467-019-09051-x (PMC6408466; doi:10.1038/s41467-019-09051-x)
Supplement: Supplementary file 3 — Description of Additional Supplementary Files [file 41467_2019_9051_MOESM3_ESM.docx]

**Title: Supplementary Movie 1**

**Description:** Swimming of wild type, *fap45*, *fap52*, and *fap45fap52* in TAP medium.

**Title: Supplementary Movie 2**

**Description:** High-speed movies of wild type, *fap45*, *fap52*, and *fap45fap52* in TAP medium.

**Title: Supplementary Movie 3**

**Description:** Swimming of confluent cultured *fap45fap52* in TAP medium.

**Title: Supplementary Movie 4**

**Description:** Swimming of wild type and *fap45fap52* in TAP with 7.5% ficol.

**Title: Supplementary Movie 5**

**Description:** HS-AFM movie of wild type DMT on a mica surface.

**Title: Supplementary Movie 6**

**Description:** HS-AFM movie of *fap45fap52* DMT on a mica surface.

**Title: Supplementary Movie 7**

**Description:** HS-AFM movie of *fap45* DMT on a mica surface.

**Title: Supplementary Movie 8**

**Description:** HS-AFM movie of *fap52* DMT on a mica surface.

**Title: Supplementary Movie 9**

**Description:** *fap20*, *fap20fap45*, *fap20fap52*, and *fap20fap45fap52* in TAP.
